# Protein energy landscapes determined by five-dimensional crystallography

**DOI:** 10.1107/S0907444913025997

**Published:** 2013-11-19

**Authors:** Marius Schmidt, Vukica Srajer, Robert Henning, Hyotcherl Ihee, Namrta Purwar, Jason Tenboer, Shailesh Tripathi

**Affiliations:** a Physics Department, University of Wisconsin-Milwaukee, Milwaukee, Wisconsin, USA; b Center for Advanced Radiation Sources, The University of Chicago, Chicago, Illinois, USA; c Center for Nanomaterials and Chemical Reactions, Institute for Basic Science, Daejeon 305-701, Republic of Korea; d Department of Chemistry, KAIST, Daejeon 305-701, Republic of Korea

**Keywords:** five-dimensional crystallography, time-resolved crystallography, time-resolved microspectrophotometry, chemical kinetics, photoactive yellow protein

## Abstract

Free-energy landscapes decisively determine the progress of enzymatically catalyzed reactions [Cornish-Bowden (2012 ▶), *Fundamentals of Enzyme Kinetics*, 4th ed.]. Time-resolved macromolecular crystallography unifies transient-state kinetics with structure determination [Moffat (2001 ▶), *Chem. Rev.* **101**, 1569–1581; Schmidt *et al.* (2005 ▶), *Methods Mol. Biol.* **305**, 115–154; Schmidt (2008 ▶), *Ultrashort Laser Pulses in Medicine and Biology*] because both can be determined from the same set of X-ray data. Here, it is demonstrated how barriers of activation can be determined solely from five-dimensional crystallo­graphy, where in addition to space and time, temperature is a variable as well [Schmidt *et al.* (2010 ▶), *Acta Cryst.* A**66**, 198–206]. Directly linking molecular structures with barriers of activation between them allows insight into the structural nature of the barrier to be gained. Comprehensive time series of crystallo­graphic data at 14 different temperature settings were analyzed and the entropy and enthalpy contributions to the barriers of activation were determined. One hundred years after the discovery of X-ray scattering, these results advance X-ray structure determination to a new frontier: the determination of energy landscapes.

## Abstract 2

Barriers of activation within the photocycle of a photoactive protein were extracted from comprehensive time courses of time resolved crystallographic data collected at multiple temperature settings.

## 1. Introduction

**Figure 1:**
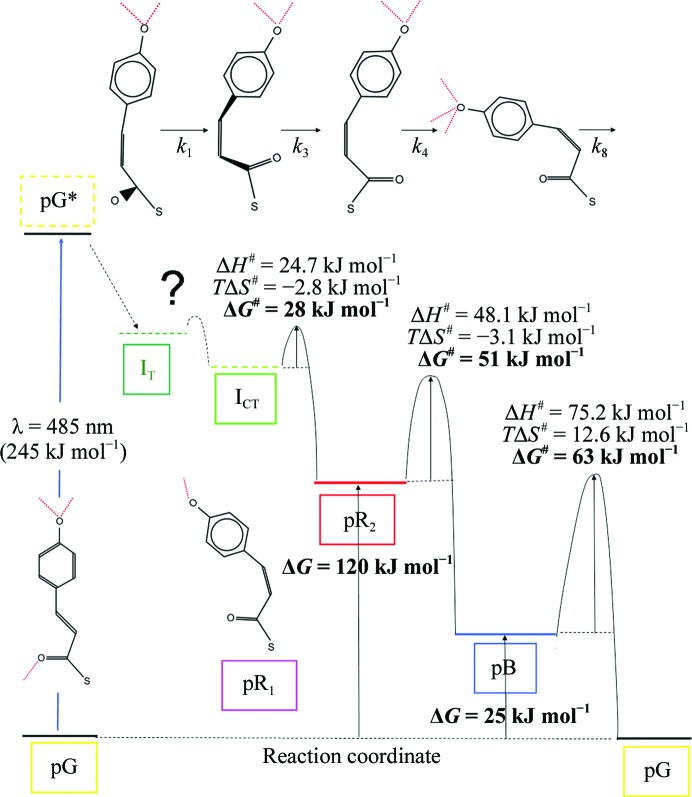
Free-energy landscape of the main reaction pathway in PYP. The reaction coordinate has periodic boundaries (pG). Atomic structures of pG, I_T_, I_CT_, pR_1_, pR_2_ and pB are known. Early transitions from pG* require higher time resolution. The energy levels of I_T_ and I_CT_ are unknown (dashed lines); the free-energy levels of pR and pB are known in solution (Takeshita *et al.*, 2002 ▶; van Brederode *et al.*, 1995 ▶). The free-energy and entropic and enthalpic contributions to the barriers as extracted from the five-dimensional crystallographic data are shown at 300 K.

**Figure 2:**
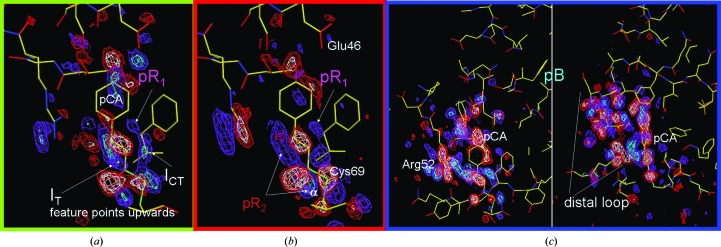
Experimental difference maps near the *p*-coumaric acid (pCA) chromophore in PYP averaged through various time intervals and at various temperatures and contoured at ±3σ in blue and red and ±4σ in cyan and white, respectively. Averaging was performed solely to enhance the appearances of the maps and to illustrate signature features of the intermediate states. (*a*) Average difference map obtained from the eight earliest difference maps from 2 to 8 ns at *T* < 263 K. Features of three intermediates, I_T_, I_CT_ and pR_1_, contribute to the same set of maps. (*b*) Average difference map obtained from 20 difference maps at *T* < 263 K from 256 ns to 64 µs. Features of two intermediates, pR_1_ and pR_2_, contribute. Feature α is the strongest in all maps at up to 12σ above the noise. It denotes the displacement of the Cys69 S atom in pR_2_. (*c*) Average difference map obtained from 20 difference maps in the millisecond time range at 303 < *T* < 323 K. Main features are found near the chromophore and on the distal loop.

**Figure 3:**
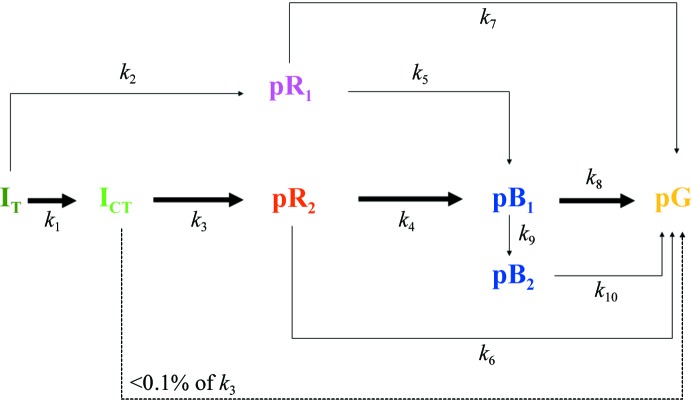
Chemical kinetic mechanisms and rate coefficients of the PYP photocycle. At temperatures up to 313 K, eight rate coefficients (*k*_1_ to *k*_8_) and five intermediate states plus the dark state contribute. The occupancy of pB_2_ is very low and cannot be observed. Above 313 K the early intermediates are not detected because the time series start around 100 ns. pB_2_ accumulates to a detectable extent and the rate coefficients *k*_9_ and *k*_10_ contribute in addition. The main reaction pathway in PYP is indicated by bold arrows. The direct path from I_CT_ to pG is irrelevant (dashed arrow).

Since the 1890s, the Van’t Hoff–Arrhenius equation, νexp(−β*E*_a_), has been used to describe the temperature dependence of chemical reaction rates. *E*_a_ is the energy of activation and the factor β = 1/(*k*_B_ *T*) containing the Boltzmann factor *k*_B_ accounts for the inverse temperature behavior. The pre-factor ν accounts for the dynamic behavior of the members of the ensemble. Eyring (1935 ▶) tied this equation to a transition state at the top of the barrier of activation, 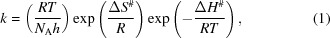
where *R* is the gas constant, *N*_A_ is Avogadro’s number, *h* is Planck’s constant and Δ*S* ^#^ and Δ*H* ^#^ are the entropy and enthalpy differences from the initial state to the transition state, respectively. A reaction can be followed with time-resolved methods, from which conclusions on the underlying mechanism are drawn by kinetic modeling. In early approaches (Gibson, 1952 ▶; Austin *et al.*, 1975 ▶), the structures of the reaction intermediates were inferred from static crystallography. Time-resolved crystallography (TRX; Moffat, 1989 ▶) finally unified kinetics with structure determination (Šrajer *et al.*, 1996 ▶; Schmidt *et al.*, 2003 ▶; Schmidt, 2008 ▶). Once the structures of intermediates are known, kinetic mechanisms can be tested by post-refinement against the TRX data (Schmidt, 2008 ▶; Schmidt *et al.*, 2004 ▶). If the temperature is varied, the previously four-dimensional crystallographic data become five-dimensional (Schmidt *et al.*, 2010 ▶). The photocycle of photoactive yellow protein (PYP) is used here as a model system from which five-dimensional crystallographic data were collected. The photocycle features distinct intermediate states, structures of which were determined earlier by picosecond and nanosecond TRX at only one temperature (Schotte *et al.*, 2012 ▶; Jung *et al.*, 2013 ▶; Ihee *et al.*, 2005 ▶; Schmidt *et al.*, 2004 ▶). Absorption of a blue photon at 485 nm provides 245 kJ mol^−1^ of energy to excite the central *p*-­coumaric acid (pCA) chromophore (Fig. 1 ▶). Part of the energy is rapidly dissipated (Martin *et al.*, 1983 ▶; Fitzpatrick *et al.*, 2012 ▶). The remaining energy is stored in an energy-rich atomic configuration (Groenhof *et al.*, 2004 ▶) labeled I_T_. The chromophore is not yet fully isomerized from *trans* to *cis* (Schotte *et al.*, 2012 ▶; van Stokkum *et al.*, 2004 ▶; Jung *et al.*, 2013 ▶). The I_T_ state is followed by two states: I_CT_ and pR_1_. I_CT_ and pR_1_ are fully *cis* and branch away from I_T_ in a volume-conserving bicycle-pedal and hula-twist reaction, respectively (Jung *et al.*, 2013 ▶). The dominant species is I_CT_. In I_CT_ the carbonyl O atom is flipped to the other side but the chromophore head is still fixed by two hydrogen bonds to amino acids Tyr42 and Glu46. In pR_1_ the chromophore head hydroxyl has lost one hydrogen bond. The entire chromophore has rotated about the chromophore axis. I_CT_ relaxes to pR_2_. This relaxation causes the Cys69 S atom to which the chromophore is bound to move significantly. The strongest difference electron-density features are found near this S atom (Fig. 2 ▶ *b*). The pR_1_ and pR_2_ states are occupied for many orders of magnitude in time. Finally, they relax to the pB state (Ihee *et al.*, 2005 ▶; Schmidt *et al.*, 2004 ▶). The pB state most likely resembles the signaling state of PYP. The chromophore head forms new hydrogen bonds to the displaced Asp52 and to an additional water that appears near the entrance to the chromophore pocket (Tripathi *et al.*, 2012 ▶). Finally, pB relaxes to the dark state (pG). Microscopic rate coefficients *k* between the intermediates plus the extent of reaction initiation specify a mechanism. The mechanism proposed by two previous TR crystallographic studies of PYP (Jung *et al.*, 2013 ▶; Ihee *et al.*, 2005 ▶) is depicted in Fig. 3 ▶. The rate coefficients of this mechanism depend on the temperature. This dependence can be described by the transition-state equation (TSE; equation 1). Other equations such as Kramer’s equation (Hanggi *et al.*, 1990 ▶), which parameterizes the pre-factors of the rate coefficients in terms of friction, are also frequently used. With this, our results would not be comparable with earlier results on PYP (Van Brederode *et al.*, 1995 ▶, 1996 ▶; Ng *et al.*, 1995 ▶), which were based on the TSE. Accordingly, we also use the TSE and express the barrier height in terms of enthalpy and entropy differences from the transition state. We demonstrate here how these thermodynamic parameters can be extracted solely from five-dimensional crystallography.

## 2. Methods

**Figure 4:**
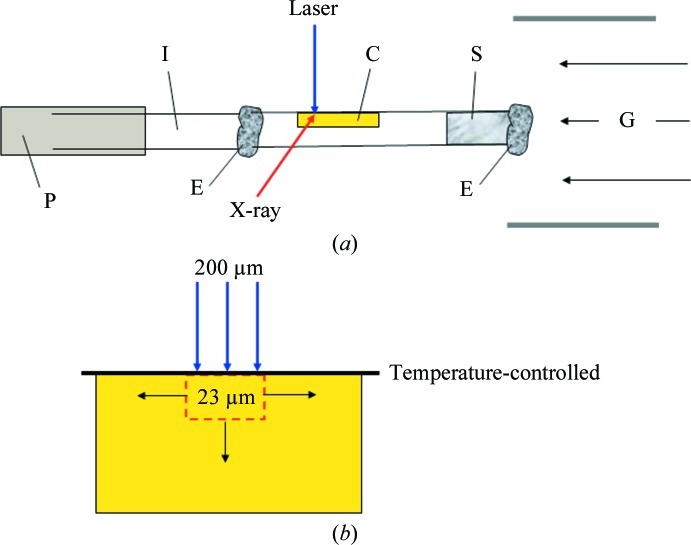
Crystal capillary mount to accommodate temperatures of >303 K. (*a*) P, brass pin; I, insulating glass capillary; E, epoxy glue; C, PYP crystal; S, stabilizing solution; G, temperature-controlled gas stream. (*b*) Laser-illuminated volume of the crystal (red dashed box). The capillary wall (thick black line) is temperature-controlled. Black arrows: heat diffuses out.

TRX experiments were conducted on crystals of PYP on the BioCARS 14-ID beamline at the Advanced Photon Source, Argonne National Laboratory, USA. Nanosecond laser pulses (∼5 ns pulse duration) from a tunable Opolette HEII laser (Opotek) were used to initiate the photocycle and the reaction was followed from nanoseconds to seconds using X-­ray pulses of ∼100 ps duration. The temperature was varied from 233 to 343 K using a Cryojet (Agilent Technologies) up to 293 K and a Cryostream (Oxford Cryosystems) for higher temperatures. PYP crystals were grown as described elsewhere (Borgstahl *et al.*, 1995 ▶). The PYP crystals were mounted in 1 mm glass capillaries glued into brass pins. For temperatures of >303 K the capillaries must be insulated from the cold brass pins, since otherwise severe distillation effects would destroy the crystals (Fig. 4 ▶ *a*). With this setup, temperatures of up to 343 K can be tolerated. The temperature at the crystal site was determined with a calibrated diode and used for subsequent calculations (Table 1 ▶). At *T* > 348 K wild-type PYP thermally denatures (Meyer *et al.*, 2003 ▶) and, accordingly, the crystals start to decompose. Within an ∼110 K temperature range (233–343 K), the photocycle was probed at 14 different temperature settings using 21–31 time points at each temperature (Table 1 ▶ and Supplementary Material^1^). The Laue data were collected with the time as the fast variable: for a certain fixed crystal orientation diffraction patterns for all time points were collected. After this, the crystal was set to another orientation and translated to expose a fresh crystal volume. The process was repeated until a complete data set had been collected. 20 different crystal orientations were used to cover reciprocal space. The data sets were processed by *Precognition*/*Epinorm* (RenzResearch; see Table 1 ▶ and Supplementary Material for data statistics). Difference electron-density maps were calculated on the absolute scale using weighted difference structure factors as described elsewhere (Tripathi *et al.*, 2012 ▶; Schmidt, 2008 ▶). The time-dependent difference maps were analyzed by singular value decomposition (SVD) using the program *SVD*4*TX* (Schmidt *et al.*, 2003 ▶; Schmidt, 2008 ▶). For the SVD, the difference maps of a time series were arranged in temporal order in data matrix **A** (see Supplementary Material for further details). Matrix **A** was subsequently decomposed into left singular vectors (lSVs) in matrix **U**, representing spatial components, and right singular vectors (rSVs), which contain the temporal information of the corresponding lSVs, in matrix **V** (2). The diagonal matrix **S** contains the singular values.
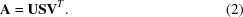
The significant rSVs at each temperature can easily be identified (see §[Sec sec3]3). All *j* significant rSVs of a time series were fitted globally by sums of exponentials, employing a common set of observable kinetic rates Λ_*i*_. Different sets of amplitudes *A*_*i*,*j*_ plus offsets *A*_0,*j*_ were used for each individual rSV (3), 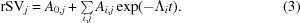
The relaxation times τ_*i*_ of the kinetic phases in the reaction are the reciprocals of Λ_*i*_. The analysis was performed for each temperature, resulting in a set of temperature-dependent observable rates and corresponding relaxation times.

To determine microscopic rate coefficients of the individual interconversions between the intermediates, kinetic modeling of the five-dimensional crystallographic data is necessary. This is accomplished using (4) and (5). The mechanism displayed in Fig. 3 ▶ was employed. Time-dependent fractional concentrations of the intermediates, *c*_*i*_(*t*, *k*), were calculated by the program *GetMech* (Schmidt *et al.*, 2004 ▶) by integrating the coupled differential equations (Steinfeld *et al.*, 1985 ▶) that describe this mechanism. The concentrations were used to generate time-dependent difference maps from the time-independent difference maps of the intermediates according to 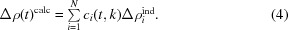
The time-independent difference maps of the intermediates Δρ_*i*_^ind^ used in (4) were calculated by subtracting the structure factors of the dark-state structure from those of the respective (known) intermediate structures and subsequent Fourier synthesis. The structures of the dark state and intermediates were obtained from the Protein Data Bank (Berman *et al.*, 2002 ▶). The nomenclature used in previous studies (Genick *et al.*, 1997 ▶; Ihee *et al.*, 2005 ▶; Kim *et al.*, 2012 ▶; Yeremenko *et al.*, 2006 ▶; Jung *et al.*, 2013 ▶) is followed here. The structures of the intermediates are, in order of appearance after reaction initiation (Fig. 3 ▶), I_T_, pR_1_, I_CT_, pR_2_, pB_1_ and pB_2_ (Ihee *et al.*, 2005 ▶; Jung *et al.*, 2013 ▶), with PDB entries 3ve3, 1ts7, 3ve4, 1ts0 and 1ts6, respectively. The structure of the dark/reference state was obtained from PDB entry 2phy (Borgstahl *et al.*, 1995 ▶). It was assumed here that the structural differences between the intermediates and the dark structure are temperature-independent. The program *GetMech* was further used to optimize the microscopic rate coefficients, *k*, of the assumed mechanism (Fig. 3 ▶). By varying the microscopic rate coefficients, the concentrations *c*_*i*_(*t,* *k*) are modified, which subsequently change the calculated time-dependent difference maps Δρ(*t*)^calc^ *via* (4). These calculated time-dependent difference maps were compared with all measured (observed) difference maps Δρ(*t*)^obs^ for each particular time series (5) and the rate coefficients were varied until convergence (Schmidt, 2008 ▶; Tripathi *et al.*, 2012 ▶; Schmidt *et al.*, 2012 ▶),
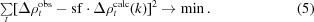
In addition, a scale factor (sf) is determined by (5) that is equal to the extent of reaction initiation. The extent of reaction initiation is equivalent to the fractional concentration of PYP molecules that are active (not in the dark state) in the crystal at the beginning of our time series. As a result, temperature-dependent microscopic rate coefficients were obtained. These were then fitted by the TSE to obtain the entropy and enthalpy differences of the barriers of activation. The programs *SVD*4*TX* and *GetMech* can be found on the web page of MS (http://users.physik.tu-muenchen.de/marius/Software.htm).

Time-resolved absorption spectra were collected from crushed PYP crystals following reaction initiation at time delays from 20 µs to a few seconds at 273 and 303 K with a home-built fast microspectrophotometer (Purwar *et al.*, 2013 ▶). Time-dependent difference absorption spectra were obtained by subtracting the time-resolved absorption spectra from that obtained from PYP in the dark. The time series were also analyzed by SVD using *MatLab* (MathWorks) routines to extract relaxation times (see Supplementary Material for further details).

## 3. Results

**Figure 5:**
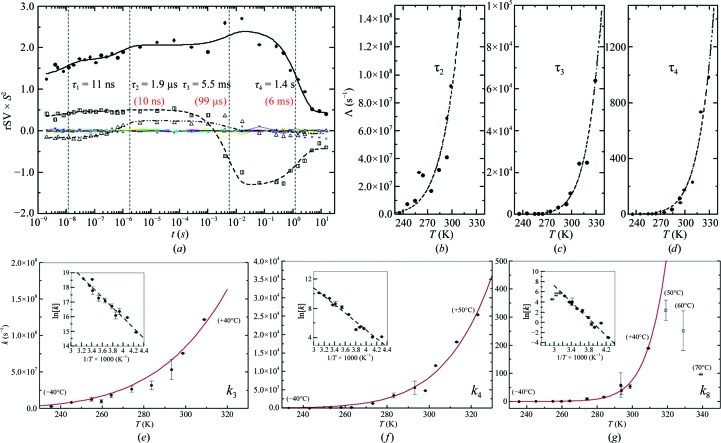
(*a*) Right singular vectors (rSVs) at 233 K. The rSVs are weighted by the square of their respective singular value *S*. Four kinetic processes τ_1_….τ_4_ are globally observed (dashed vertical lines). Solid spheres, open triangles and squares: first, second and third significant rSVs. Colored thin lines around zero: remaining less significant rSVs. Solid black line, dashed line and dashed double dotted line: global fit of the significant rSVs by four exponential functions with the same set of relaxation times but different amplitudes. Relaxation times obtained at room temperature (298 K, red) are shown in parentheses for comparison. (*b*), (*c*) and (*d*): macroscopic rates Λ (inverse of relaxation times) for processes τ_2_, τ_3_ and τ_4_ plotted as a function of temperature, respectively. Dashed lines: fits by the Van’t Hoff–Arrhenius equation. (*e*), (*f*) and (*g*): temperature dependence of the main pathway microscopic rate coefficients *k*_3_, *k*_4_ and *k*_8_, respectively. Red lines, fits by the TSE. Insets, Arrhenius plots: dashed lines, fits by straight lines.

**Figure 6:**
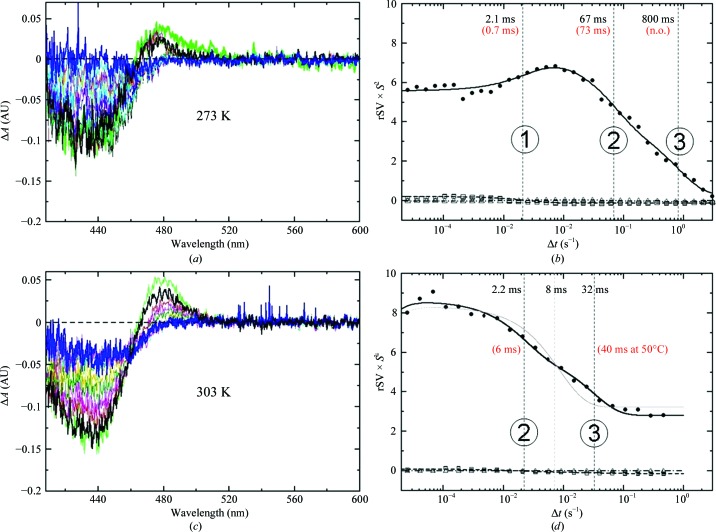
Time-resolved difference absorption spectra of crystalline slurry at 273 K (*a*) and 303 K (*c*) (black, earliest time point; blue, last time point). (*b*, *d*) Right singular vectors (rSVs) from SVD analysis of the difference spectra. Solid lines, global fit by sums of three exponentials identifying three processes. Processes 1–3 are labeled with their respective relaxation times and are marked with thin vertical lines; relaxation times from crystallography are shown in red brackets (n.o., not observed). The gray line in (*d*) is the fit of the final relaxation with only one exponential.

Table 1 ▶ gives an overview of the collected Laue data (also see Supplementary Material). Since the recent upgrade of the BioCARS 14-ID beamline (Graber *et al.*, 2011 ▶), Laue data of excellent quality with *R*_merge_ in the range of 5% and *I*/σ(*I*) > 20 can be collected rapidly (Tripathi *et al.*, 2012 ▶; Schmidt *et al.*, 2012 ▶), which makes comprehensive studies such as this possible. Fig. 5 ▶(*a*) shows an example of right singular vectors (rSVs) extracted from the TRX data by SVD (Schmidt *et al.*, 2003 ▶). The right singular values are exquisitely smooth owing to the excellent data quality and data-collection strategies at BioCARS (Graber *et al.*, 2011 ▶). As shown previously (Schmidt *et al.*, 2012 ▶), an entire time series can be collected from a single PYP crystal; the absorbed X-ray dose and damage inflicted by the laser is below the kinetic dose limit *D*_K_^1/2^ for PYP. In this way, the crystal-to-crystal scaling variations which plagued earlier investigations (Ihee *et al.*, 2005 ▶; Rajagopal *et al.*, 2005 ▶; Schmidt, Nienhaus *et al.*, 2005 ▶) are avoided. As a result, although rSVs 1–15 are displayed in Fig. 5 ▶(*a*), the less significant rSVs (4–15) are distributed closely around zero (colored thin lines in Fig. 5 ▶ *a*). A global fit with four exponentials identifies four kinetic processes Λ_1_…Λ_4_ with relaxation times τ_1_…τ_4_. The process with relaxation time τ_1_ results from the nonzero laser pulse width and the decay of I_T_ to both I_CT_ and pR_1_ that can be identified in the earliest difference maps. Processes τ_2_ to τ_4_ result from relaxations of states I_CT_ to pR_2_ (τ_2_), the joint relaxations of pR_1_ and pR_2_ to pB and pG (τ_3_) and finally from pB to pG (τ_4_), respectively. These processes accelerate when the temperature is increased (see Fig. 5 ▶ *a*, numbers shown in red). The rSVs as well as the relaxation times at all temperatures can be found in Supplementary Fig. S3. In Figs. 5 ▶(*b*)–5 ▶(*d*), the relaxation rates Λ_i_ are plotted as a function of temperature and fitted by the Van’t Hoff–Arrhenius equation (dashed lines). The pre-factors and the energies of activation *E*_a_ derived from the fits are shown in Table 2 ▶. Slower relaxation times from TRX and time-resolved microspectroscopy (TRS) agree reasonably (Fig. 6 ▶ and Table 2 ▶). At 273 K three processes are observed in TRS. Process (1) corresponds to the pR to pB transition. The relaxation time derived from TRS is 2.1 ms, which compares with process τ_3_ (0.7 ms) obtained from TRX. The pB to pG transition is biphasic (processes 2 and 3). The relaxation time of process (2) is 67 ms at 273 K, compared with τ_4_ = 73 ms obtained from TRX, and 2.2 ms at 303 K, compared with τ_4_ = 6 ms observed crystallographically. Process (3) observed with TRS at 273 K contributes in a minor way, so that it cannot be detected with TRX. However, at elevated temperatures it can also be detected by crystallography with a similar relaxation time. Notably, the photocycle can be observed crystallo­graphically up to 343 K and PYP also remains active at low temperatures (233 K) where the photocycle completes in ∼10 s.

The main reaction pathway through pR_2_ follows microscopic rate coefficients *k*_1_, *k*_3_, *k*_4_ and *k*_8_ (compare Figs. 1 ▶ and 3 ▶). A minor pathway involves pR_1_ which branches away from I_T_. Rate coefficient *k*_7_ is generally 50% of *k*_5_. One third of the pR_1_ molecules relax directly to pG, while two thirds populate pB. Since the pR_1_ occupancy is low, the rate coefficient *k*_7_ is difficult to determine and therefore may vary substantially. pR_2_ typically decays mainly to pB; *k*_6_ is generally much smaller than *k*_4_. In earlier PYP studies (Ihee *et al.*, 2005 ▶; Tripathi *et al.*, 2012 ▶) the pathways through *k*_6_ and *k*_7_ were not taken into account. Here we considered them as well as a more general possibility. Both pathways add little to the mechanism, since the pR_1_ occupancy is small and only a small fraction of pR_2_ relaxes directly to pG. The main product is pB, which decays with rate *k*_8_. At higher temperatures only rate coefficients *k*_4_ to *k*_10_ were included. This mechanism lacks the early intermediates as their population decays rapidly compared with the earliest time delay. It features in addition two scale factors that account for the amount of pR_1_ and pR_2_ and an extra state pB_2_. The two pR states are fully occupied after 20 ns at these temperatures (Jung *et al.*, 2013 ▶) and relax to pB on the microsecond time scale. At 323 K a weak second pB phase appears that indicates the presence of pB_2_ (see Supplementary Fig. S3). Rather than speeding up, the photocycle slows down (Fig. 5 ▶ *g*) because the PYP occupies additional pB-like states even in the crystal. The concentration profiles of the intermediates closely display the relaxation times observed in the rSVs (see also Supplementary Fig. S3).

For all rate coefficients, except for *k*_1_ and *k*_2_, where we are limited by the time resolution of the experiment, thermal activation is observed. The photocycle accelerates by a factor of 318 when the temperature is changed by 80 K (from 1.4 s at 233 K to 4.4 ms at 313 K). This agrees nicely with the well known *Q*_10_ rule, which predicts that the reaction velocity in enzymes doubles when the temperature increases by 10 K. The *Q*_10_ rule predicts an acceleration by a factor of 256 (2^8^) compared with the factor of 318 observed here. Above 323 K PYP surpasses its temperature optimum and the photocycle slows down again (Fig. 5 ▶ *g*). Therefore, the rate coefficients extracted at 333 and 343 K were not included in the fit of the TSE. In Figs. 5 ▶(*e*)–5 ▶(*g*) the temperature dependences of *k*_3_, *k*_4_ and *k*_8_ are shown (those of *k*_5_ and *k*_7_ are shown in the Supplementary Material). The barrier height varies from 27.5 kJ mol^−1^ for I_CT_ decay (*k*_3_) to 63 kJ mol^−1^ for pB_1_ depopulation (*k*_8_) (Fig. 1 ▶ and Table 2 ▶). Absorption of the laser pulse deposits energy into a volume approximately determined by the laser footprint on the crystal, the crystal diameter and the penetration depth (Fig. 4 ▶ *b*). An adiabatic temperature jump of about 11 K is estimated (see Supplementary Material). By correcting for the jump, the barriers shift by only ∼1.5 kJ mol^−1^ and the entropic contributions remain almost the same. A portion of the absorbed energy is initially stored in the twisted chromophore geometry of I_T_ and is only released gradually through exothermic relaxation processes. Owing to the shallow penetration depth of the laser, the heat dissipates rapidly on a submillisecond time scale. The final pB to pG relaxation is never affected. Accordingly, we report uncorrected values here.

## 4. Discussion

The mechanism employed (Fig. 3 ▶) was motivated by earlier TRX experiments with nanosecond (Ihee *et al.*, 2005 ▶) and picosecond (Jung *et al.*, 2013 ▶) time resolution using PYP crystals grown as originally described in Borgstahl *et al.* (1995 ▶). Another picosecond TRX experiment was recently performed (Schotte *et al.*, 2012 ▶) on different PYP crystals which were grown in heavy water and at high salt concentrations (1.1 *M* NaCl, pD 9) and that were intended to be used in neutron diffraction experiments. Between these two approaches, there are very subtle differences in the intermediate structures on fast time scales and two major differences on longer time scales. (i) The final relaxation time from pB to pG observed by Schotte and coworkers at 288 K is much longer (∼260 ms) than that of ∼20 ms observed by Ihee and corworkers and also by us. (ii) Schotte and coworkers did not observe the intermediate that we call pR_1_ here and interpret their 10 ns–100 µs time range using only a single pR_2_-like structure. Hence, the pathway through rate coefficients* k*_2_, *k*_5_ and *k*_7_ (Fig. 3 ▶) was not included in their mechanism. This pathway must be added to our mechanism, since pR_1_ is observed in our difference maps at the earliest times (Fig. 2 ▶ *a*) and continues to be occupied into the long microsecond time range until it decays simultaneously with pR_2_. The most likely reason for the differences in the detailed kinetic mechanism and altered relaxation times of Schotte and coworkers is that their PYP crystals were grown under quite different conditions (heavy water and high salt; see above). Even when the pH is shifted, a previous pH-dependent TRX study with the same type of crystals that we used (Tripathi *et al.*, 2012 ▶) shows that pR_1_ is present at all pH values (pH 4, 7 and 9) and decays in concert with pR_2_. Since we knew that the external conditions such as the pH and salt concentration can alter the mechanism (Tripathi *et al.*, 2012 ▶; Borucki *et al.*, 2005 ▶, 2006 ▶), we used previously established uniform crystallization conditions throughout and we very carefully adjusted the pH to 7 for our study.

The TSE describes the temperature dependence of the rate coefficients *k* in the range from 233 to 323 K. This makes it possible to infer barriers of activation in the PYP photocycle in the crystal (Fig. 1 ▶). In Fig. 1 ▶ we adopted some of the conformational free energies from solution (Takeshita *et al.*, 2002 ▶; van Brederode *et al.*, 1995 ▶) with the strong caveat that they might be very different in the crystal. Barriers of activation from TRX are shown for the main reaction pathway. The final barrier is the rate-limiting step of the reaction. It slows down the photocycle decisively so that the signaling state may persist. The chromophore pocket opens to the solvent and becomes exceptionally susceptible to additional stimuli such as the pH (Tripathi *et al.*, 2012 ▶; Borucki *et al.*, 2006 ▶).

Fitting the Van’t Hoff–Arrhenius equation to macroscopic rates, Λ, yields apparent energies of activation *E*_a_ of the corresponding observable kinetic phases. However, these phases result from a number of underlying interconversions between intermediate states determined by microscopic rate coefficients of the mechanism in Fig. 3 ▶. Therefore, the *E*_a_ values in Table 2 ▶ are not, or are only approximately, meaningful for individual interconversions, and a chemical kinetic mechanism is needed. Within the constraints of the mechanism employed, the TSE allows the separation of entropic and enthalpic contributions to the barriers from the temperature-dependent microscopic rate coefficients (Fig. 1 ▶). PYP intermediates occupy minima on the free-energy surface because the chromophore is immersed into a tight hydrogen-bonding network. Δ*H* ^#^ is positive because for the reaction to occur some of the bonds have to be broken. Δ*S* ^#^ reflects the gain or loss of degrees of freedom. If the protein has time to relax, the structure can fluctuate through the substates and the entropy change is positive (Parak *et al.*, 2007 ▶). If, however, the protein environment stays rigid, there might be only one well defined narrow path for the transition. In this case Δ*S* ^#^ may become negative. By inspecting the structures of the intermediates, the structural reasons for the observed values become clear. However, the structures of the transition states themselves remain unknown because their occupation is minute. With a Δ*G* ^#^ of 63 kJ mol^−1^ for the pB to pG transition, for example, the probability of catching a molecule on the top of the barrier at 300 K is only exp(−Δ*G* ^#^/*RT*) = 1.1 × 10^−11^. In a macroscopic crystal with about 10^14^ molecules, about 1000 molecules are on top of a barrier at any given time. Such low occupancy cannot be detected. However, the structures of the intermediate states that flank the barrier can be determined and the nature of the barrier can be inferred from them. For the pR_2_ to pB transition, for example, the chromophore lifts out of a hydrogen-bonding network involving Tyr42, Glu46 and Cys69. Δ*H* ^#^ is positive. The chromophore then rotates. On the microsecond time scale protein relaxations are incomplete, as is evident from the absence of extensive features in difference electron-density maps except in the direct vicinity of the chromophore (Figs. 2 ▶ *a* and 2 ▶ *b*). Consequently, there is only limited space for this rotation and the entropic contribution to the barrier is slightly negative. The situation is different for the pB to pG transition. Once the hydrogen bonds of the pCA head hydroxyl to Arg52 and to one or two water molecules break, the pCA can re-isomerize back to *trans*. At these longer millisecond to second time scales the protein structure is relaxed, as is obvious from numerous difference electron-density features on protein moieties surrounding the chromophore pocket (Fig. 2 ▶ *c*). Once the pCA head is free, it can form a transition state that occupies an enlarged, relaxed chromophore pocket. Δ*S* ^#^ becomes positive (Table 1 ▶). The entropic contribution helps to accelerate the reaction by lowering the barrier. In solution the PYP structure relaxes even further. For the pB to pG transition Δ*H* ^#^ is only 10 kJ mol^−1^ at pH 3 and *T*Δ*S* ^#^ is largely negative at −60 kJ mol^−1^ (Van Brederode *et al.*, 1996 ▶). In solution re-isomerization is controlled almost entirely by the entropy and PYP refolds *via* a transition state which is much more ordered than the pB intermediate. The structures of both the highly unfolded intermediate and the transition state remain elusive. In the crystal, however, the entropy plays a smaller role because a highly unfolded intermediate (as in solution) apparently does not form.

The PYP photocycle is an excellent model to study macromolecular reactions and to develop new methodologies that can be generalized to the investigation of other proteins and enzymes. By lowering the temperature to below 273 K reactions slow down so that intermediates such as I_T_ (and I_CT_) that were previously observed only with picosecond time-resolved crystallography (Jung *et al.*, 2013 ▶; Schotte *et al.*, 2012 ▶) become observable on the nanosecond time scale. Barriers of activation of reactions that occur within biological macromolecules can be determined from time-resolved crystallo­graphic data alone. When pharmaceutically relevant enzymatic reactions are investigated each intermediate structure can be a potential drug target. However, the determination of transient structures in enzymes is more difficult because their reactions are noncyclic or irreversible. Free-energy landscapes including barriers of activation are decisive for their function. It is therefore desirable to develop methods to rapidly and routinely investigate these reactions on the near-atomic length scale by TR methods at the synchrotron or potentially at the X-ray free-electron laser with microcrystals and nanocrystals (Schmidt, 2013 ▶; Aquila *et al.*, 2012 ▶; Neutze & Moffat, 2012 ▶) or even in solution (Poon *et al.*, 2013 ▶). In solution, as well as in very small crystals, diffusion times are short. This enables rapid reaction initiation by simply mixing the crystals or solution with substrate and injecting the mixture into the X-ray beam (Schmidt, 2013 ▶). Photosensitivity has been engineered genetically into protein structures (Möglich *et al.*, 2010 ▶), and specially designed and manufactured caged substrates (Goelder & Givens, 2005 ▶) are also used for convenient reaction initiation by laser pulses. These methods, in combination with new and existing powerful X-ray sources, will be available to explore free-energy surfaces and investigate a multitude of enzymatic reactions with five-dimensional crystallography.

## 5. Conclusions

One hundred years after X-ray diffraction was discovered, we show how crystallography can be used to determine barriers of activation in biological macromolecules. Our findings obtained solely from crystallography demonstrate that from 233 to 323 K all processes in PYP are thermally activated and show approximate Arrhenius behavior. Above 323 K the reaction surpasses its temperature optimum and slows down again. Meaningful thermodynamic parameters that describe barriers of activation in the PYP photocycle can be extracted and structurally understood.

## Supplementary Material

Supplementary material file. DOI: 10.1107/S0907444913025997/dw5067sup1.pdf

## Figures and Tables

**Table 1:** Statistics for selected Laue data sets at selected temperatures (for all temperatures, see Supplementary Material) *T*_set_ is the temperature set by the temperature controller of the cryogenic gas jet. *T*_c_ is the actual temperature measured by the calibrated diode at the crystal site. The time delays shown are the delays between the peak of the laser pulse to the rising edge of the X-ray pulse. The completeness of the Laue data was calculated including singlet and deconvoluted harmonic reflections. *R*_merge_ is calculated from singlet intensities using multiple measurements and symmetry equivalents. Both completeness and *R*_merge_ are given for the dark data set (the statistics are comparable for light data sets). Values in parentheses are for the last resolution shell (1.9–1.8 Å). *R*_scale_ is calculated from amplitudes (*F*) after scaling the time-resolved structure-factor amplitudes *F* ^Δ*t*^ to calculated dark *F* ^D^ amplitudes (on the absolute scale). Δρ_min_/σ_Δρ_ and Δρ_max_/σ_Δρ_ are the most negative and most positive difference electron-density features in units of the σ level found in the difference map at a selected time point (Δ*t*). The largest features can be found at and near the S atom of Cys69.

*T*_set_ (K)	*T*_c_ (K)	Time points	Completeness (%)	*I*/σ(*I*)	*R*_merge_ † (%)	*R*_scale_, Δ*t* ‡ (%)	Δρ_min_/σ_Δρ_ and Δρ_max_/σ_Δρ_, Δ*t*
233	235.5	31, 2 ns–15 s	82.5 (75.9)	16.9 (11.0)	8.3	7.7, 3 µs	−6/+10, 3 µs
263	259.5	29, 2 ns–8 s	88.0 (82.3)	23.0 (18.5)	6.8	6.9, 4 µs	−7/+9, 4 µs
283	283.6	27, 2 ns–128 ms	82.2 (77.8)	30.3 (26.9)	5.2	3.9, 4 µs	−10/+11, 4 µs
303	298.5	27, 2 ns–128 ms	83.5 (75.9)	25.3 (16.1)	5.6	6.8, 4 µs	−11/+10, 4 µs
343	338.8	21, 100 ns–1 s	84.6 (80.3)	30.6 (21.7)	4.7	4.4, 800 ns	−6/+6, 800 ns

† *R*_merge_ = 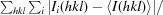 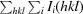.

‡ *R*_scale_ = 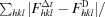 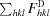.

**(a):** Macroscopic observable rates Λ_*i*_ from TR crystallography. Temperature dependences are fitted by the Van’t Hoff–Arrhenius equation. The temperature dependence of process τ_1_ (Λ_1_) cannot be determined owing to limited time resolution. n.a., not applicable.

Macroscopic rate coefficients	Λ_1_	Λ_2_	Λ_3_	Λ_4_
Pre-factor ν (s^−1^)	n.a.	1.6 × 10^14^	1.9 × 10^13^	7.7 × 10^10^
Energy of activation *E*_a_ (kJ mol^−1^)	n.a.	35.9	53.4	49.6

**(b):** Energetics derived from fitting the TSE to the temperature dependence of selected microscopic rate coefficients (the errors from the fit are given in parentheses).

Microscopic rate coefficients	*k*_3_ †	*k*_4_ †	*k*_5_ †	*k*_8_
Δ*H* ^#^ (kJ mol^−1^)	24.7	48.1	50.0	75.2 (0.03)
Δ*S* ^#^ (J mol^−1^ K^−1^)	−9.4	−10.2	−14.8	41.9 (0.08)
*T*Δ*S* ^#^ ‡ (kJ mol^−1^)	−2.8	−3.1	−4.5	12.6 (0.02)
Δ*G* ^#^ ‡ (kJ mol^−1^)	27.5	51.2	54.5	62.7 (0.09)

**(c):** Comparison of processes (1) to (3) observed by TRS with those derived from TRX (τ_3_ and τ_4_). An extra phase (3) is observed by TRX only at elevated temperatures. n.o., not observed.

Processes observed	(1)/τ_3_	(2)/τ_4_	(3)
273 K			
TRS	2.1 ms	67 ms	800 ms
TRX	0.7 ms	70 ms	n.o.
303 K			
TRS	n.o.	2.2 ms	32 ms
TRX	99 µs	6 ms	40 ms (323 K)

† The errors of the fitted parameters are smaller than 1%.

‡ At 300 K.

## Footnotes

1 Supplementary material has been deposited in the IUCr electronic archive (Reference: DW5067).
