# Supplementary material for: Protein energy landscapes determined by five-dimensional crystallography
Source: Acta Crystallogr D Biol Crystallogr. 2013 Nov 19;69(Pt 12):2534–42. doi: 10.1107/S0907444913025997 (PMC3852658; doi:10.1107/S0907444913025997)
Supplement: Supplementary file 1 [file d-69-02534-sup1.pdf]

Protein Energy Landscapes Determined by 5-Dimensional Crystallography

Marius Schmidt[a], Vukica Srajer[b], Robert Henning[b], Hyotcherl Ihee[c,d], Namrta Purwar[a], Jason Tenboer[a], Shailesh Tripathi[a]

[a]Physics Department, University of Wisconsin-Milwaukee, Milwaukee, WI, USA,

[b]Center for Advanced Radiation Sources, The University of Chicago, Chicago, IL, USA

[c]Center for Nanomaterials and Chemical Reactions, Institute for Basic Science, Daejeon, 305-701, Republic of Korea

[d]Department of Chemistry, KAIST, Daejeon, 305-701, Republic of Korea

Supplementary Material

The supplemental material reports details that the authors consider not vital to be included in the main text but worth reporting. The supplementary material is not meant to be a replacement for the main text but rather be an extension that is only meaningful in combination with the main text.

Methods

Crystals and Data Collection:

Typical crystal sizes used were 100 x 100 x 700 μm^3. The laser light was focused at the crystal to a round focal spot of 200 μm, with typical pulse energy of about 4 mJ/mm^2. The reaction was followed by a series of Laue diffraction snapshots at various time delays between the ~5 ns pump (laser) and 100 ps probe (X-ray) pulses (see Tab. S1). Depending on the crystal size, three to seven pump-probe pulse sequences were accumulated prior to detector readout to obtain high-quality diffraction patterns. The waiting time between the pulse sequences, necessary for the dark

Table S1. Statistics for selected data sets at all respective temperatures. Time-delays shown are delays between the peak of the laser pulse to the rising edge of the X-ray pulse. Completeness of Laue data is calculated including single and deconvoluted harmonic reflections, R_{merge} is calculated from singlet intensities using multiple measurements and symmetry equivalents; both completeness and R_{merge} are given exemplary for the dark dataset, last shell is from 1.9 - 1.8 Å; R_{scale} is calculated from amplitudes (F) after scaling the time-resolved structure factor amplitudes $F^{\Delta t}$ to calculated dark F^D amplitudes (on the absolute scale); $\Delta\rho_{min}/\sigma_{\Delta\rho}$ and $\Delta\rho_{max}/\sigma_{\Delta\rho}$ are the most negative and most positive difference electron density features in units of the sigma level found in the difference map at a selected time point Δt. The largest features can be found at and near the sulfur atom of Cys69.

T [K]	time-points from-to	completeness (last shell)	I/σ_I (last shell)	R_{merge} (%)[a]	R_{scale} (%) @Δt[b]	$\Delta\rho_{min}/\sigma_{\Delta\rho}$ $\Delta\rho_{max}/\sigma_{\Delta\rho}$ @Δt
235.5	31 2 ns - 15 s	82.5 (75.9)	16.9 (11.0)	8.3	7.7 (3 µs)	-6/+10 (3 µs)
245.1	27 1 ns - 4 s	77.3 (73.4)	28.3 (21.8)	5.3	6.8 (2 µs)	-11/+13 (2 µs)
254.7	34 2 ns - 8 s	82.7 (78.2)	29.3 (21.4)	4.9	5.4 (4 µs)	-11/+11 (4 µs)
259.5	29 2 ns - 8 s	88.0 (82.3)	23.0 (18.5)	6.8	6.9 (4 µs)	-7/+9 (4 µs)
264.3	23 4 ns - 8 s	71.5 (66.2)	27.4 (24.8)	5.0	4.8 (4 µs)	-9/+11 (4 µs)
274.0	28 8 ns - 512 ms	75.1 (68.0)	27.1 (18.8)	5.4	5.7 (4 µs)	-9/+9 (4 µs)
283.6	27 2 ns - 128 ms	82.2 (77.8)	30.3 (26.9)	5.2	3.9 (4 µs)	-10/+11 (4 µs)
293.2	27 2 ns - 128 ms	81.7 (75.3)	20.1 (14.9)	6.8	6.9 (4 µs)	-7/+7 (4 µs)
293.7	27 2 ns - 512 ms	78.3 (73.4)	25.5 (20.0)	5.3	5.2 (4 µs)	-11/+12 (4 µs)
298.5	27 2 ns - 128 ms	83.5(75.9)	25.3 (16.1)	5.6	6.8 (4 µs)	-11/+10 (4 µs)
308.9	24 2 ns - 64 ms	84.7 (80.3	30.6 (21.7)	4.7	7.1 (4 µs)	-9/+10 (4 µs)
318.9	22 100 ns-256 ms	77.0 (73.0)	31.3 (24.8)	4.6	4.8 (4 µs)	-9/+9 (4 µs)
328.8	24 100 ns - 1 s	84.5 (79.3)	30.0 (18.1)	4.8	5.6 (2 µs)	-8/+9 (2 µs)
338.8	21 100 ns - 1 s	84.6 (80.3)	30.6 (21.7)	4.7	4.4 (800 ns)	-6/+6 (800 ns)

[a] $R_{merge} = \left[\sum_{hkl} \sum_{j} \left| I_{hkl,j} - \langle I_{hkl} \rangle \right| \right] / \left[\sum_{hkl} \sum_{j} I_{hkl,j} \right]$ [b] $R_{scale} = \left[\sum_{hkl} \left| F_{hkl}^{\Delta t} - F_{hkl}^{D} \right| \right] / \sum_{hkl} F_{hkl}^{D}$

state recovery, varied between 1 s at higher temperatures and around 20 s at lowest temperatures.

Single 100ps X-ray pulses were isolated as described (Graber *et al.*, 2011). The X-ray beam was

focused to a size of 60 μm vertically (v) and 90 μm horizontally (h) and each 100 ps pulse contained about 4 × 10^{10} photons in the hybrid mode of the APS storage ring or 10^{10} photons in the 24-bunch mode. At a particular crystal orientation we only probed by X-rays the crystal surface layer that was facing the laser (Graber *et al.*, 2011). To precisely position this layer in the X-ray beam, crystal edge scan was done where a series of weak diffraction images were collected while the crystal edge was translated through the X-ray beam. With this, the overlap of the laser-excited volume with the X-ray probed volume is optimized.

Preparation of Data Matrix A

The time-series of the difference maps were analyzed at each temperature by Singular Value Decomposition (SVD) as described in the main text. A more extensive description can be found in the literature (Schmidt *et al.*, 2003, Schmidt, 2008, Tripathi *et al.*, 2012, Schmidt, Ihee, *et al.*, 2005). To prepare data matrix A, a volume that covers an extended region of the chromophore pocket (21 amino acid residues) was masked out. The mask included Cys69, Tyr42, Ala44-Asp53, Asp65-Asp71, Phe96, Met100 and three water molecules close to the entrance of the chromophore pocket. The mask was further modified by using for the SVD analysis only grid points above or below plus or minus 3 σ, respectively, in at least one of the difference maps of each time series. The difference electron density values within this mask were arranged in temporal order and subjected to SVD. This procedure ensures that only those regions where strong signal is present are subjected to SVD. Other regions that contain low signal contribute mainly noise to the analysis and were excluded this way. After the SVD analysis (Eq. 2 in the main text), the spatial components, which are difference electron densities, are separated into the

significant left singular vectors (lSVs). The kinetics can be found in the corresponding significant right singular vectors (rSVs).

Details for Fitting a Chemical Kinetic Mechanism

Kinetic modeling is required to explain the time-dependent variations of the difference electron density values in terms of concentrations of the intermediates. All structures of the PYP photocycle intermediates in the time-range analyzed are known (Borgstahl *et al.*, 1995, Ihee *et al.*, 2005, Schmidt *et al.*, 2004, Jung *et al.*, 2013). Each structure represents a transient (=short lived) state. Up to 40° C the states are arranged in the chemical kinetic mechanism shown in Fig. 4 based on known intermediates and including all forward-going transitions. At 50° and higher the fast processes are not reliably observed. The mechanism lacks the early intermediates I_T and I_{CT}, and it features in addition state pB_2 that appears as an additional phase in the rSVs. The microscopic rate coefficients k define interconversions between the intermediate states (Fig. 3, main text). Time-dependent difference electron density features typically follow exponential functions with amplitude A_i and characteristic times τ_i (relaxation times, or their reciprocals, relaxation rates Λ_i), each for a particular intermediate i. Both, the amplitudes and the relaxation times are linear combinations of the microscopic rate coefficients of the underlying chemical, kinetic mechanism (Steinfeld *et al.*, 1985, Cornish-Bowden, 2012). These relaxation times are observed globally as kinetic processes in the rSVs. However, if relaxation times are similar, they appear as only one process. This is why only 4 processes are observed in our rSVs, but 5 intermediates contribute. The amplitudes are equivalent to (fractional) concentrations, and the relaxation times determine the variations of the concentrations with time. On the absolute scale, difference electron densities are directly proportional to concentrations (Eq. 4, main text). In

certain instances (Schmidt, Nienhaus, *et al.*, 2005) one can account for the electrons in a particular density feature and infer from this the occupancy, the fractional concentration, or even the concentration proper, of a molecule in the unit cell. In other instances the entire difference electron density of the whole unit cell can be fit by calculated difference electron density maps from chemically plausible structural models. This is in contrast to, for example, absorption spectroscopy where there are linear factors between concentration and absorption, namely the absorption coefficients that are all a-priori unknown for the intermediate state (van Stokkum *et al.*, 2004, Yeremenko *et al.*, 2006, Khoroshyy *et al.*, 2013). Initially determined absorption coefficients will yield self-consistent results, and may persist in the literature. This problem is considerably smaller in crystallography, because crystallographic data can be always represented on the absolute scale. A chemical kinetic mechanism compatible with the X-ray data must generate time-dependent concentrations that are directly commensurable with the observed difference electron density values. The mechanism becomes testable and, in certain instances excluded by posterior analysis (Schmidt *et al.*, 2003, Schmidt, 2008, Ihee *et al.*, 2005). Still, a number of mechanisms can fit the data reasonably well, and consequently may be indistinguishable or degenerate (Schmidt *et al.*, 2010). Here, however, we are not concerned with lifting this degeneracy but with extracting meaningful thermodynamic parameters within the constraints of a plausible candidate mechanism used previously with TRX data to extract intermediates (Jung *et al.*, 2013, Ihee *et al.*, 2005).

Posterior analysis allows the refinement (Eq. 5, main text) of the microscopic rate coefficients of a mechanism (Schmidt *et al.*, 2004, Jung *et al.*, 2013, Rajagopal *et al.*, 2005, Ihee *et al.*, 2005). This approach has the potential to exclude reaction pathways. This is the case when the

refinement yields rate coefficients that are so small that they may be ignored. An example is shown in Fig. 3 (main text), where the rate coefficient of the dashed pathway is less than 0.1% of k_3 throughout and, therefore, less than 0.1% of the molecules react through this pathway. Within the scope of chemical kinetics, one can estimate the number of observables present in time-resolved X-ray data and compare these to the number of fit parameters in a mechanism such as the one used here. The time-dependent concentrations of each individual intermediate follow sums of exponentials (Steinfeld *et al.*, 1985), which is reflected by the time-dependent difference electron density values following sums of exponentials as well. Each individual exponential of that sum is a kinetic phase (sometimes also called a transient, i.e. a short decay phase) of that particular intermediate. If there are N intermediates in the mechanism there are N kinetic phases for each single intermediate. Each kinetic phase follows a single exponential with amplitude A_i and a characteristic (relaxation) time τ_i. The relaxation times are common for corresponding kinetic phases of all intermediates, but the amplitude of each kinetic phase A_i differs for each intermediate. This results in N^2 observable amplitudes (although some may be as low as zero). The set of common relaxation times τ_i are determined globally from the rSVs as described above, and each kinetic phase is observable in the time-dependent difference electron density maps. Hence there are up to $N^2 + N$ observables, N common relaxation times and up to N^2 corresponding amplitudes for N intermediates. If the amplitudes of the transients are close to zero, because that particular intermediate has decayed, these kinetic phases do not contribute to the total number of observables. In the (typical) case that each of the N intermediates has only one kinetic phase (it decays to zero with a single exponential) there are only N amplitudes and N relaxation times. Hence the minimum number of observables is 2N.

The minimum number of 2N observables is faced with a number of free fit parameters in Eq. 5 (main text). The fit parameters are the microscopic rate coefficients of the mechanism whose magnitudes determine both the relaxation times and the amplitudes of the kinetic phases. Up to -10° C rate coefficients k_1 and k_2 were free fit parameter. From 0 ^{o}C k_1 was fixed to 2×10^9 1/s, and changed to 3×10^9 1/s from 25 ^{o}C (Tab. S2). k_2 was varied freely, because for a given k_1, the magnitude of k_2 accounts for the concentration of pR_1 relative to I_{CT}. Since the concentration of pR_1 is directly observable in the difference maps, k_2 can be determined. At lower temperatures pB_2 is not observed and 8 microscopic rate-coefficients (k_1 ... k_8) are fit parameters in the mechanism that connects 5 intermediate states and the dark state. One additional fit parameter is the extent of reaction initiation, which is the scale factor sf in Eq. 5 (main text). Hence, there are 9 free parameters. Since the time-course contains 5 intermediate states, the (lowest) number of observables is 10 (see above argumentation). A least squares fit of the mechanism to the data is possible at all temperatures $\leq$ 40 ^{o}C. A ninth rate coefficients (a 10th fit parameter) spanning from I_{CT} directly to pG can be included and its magnitude determined. This rate coefficient is smaller than 0.1% of k_3 so that the pathway is irrelevant within the constraints of the mechanism. Also k_6 is much smaller than k_4 throughout (see Tab. S2). As a consequence, the fitted values of k_6 are fluctuating largely and contribute little to the mechanism. This pathway can also be ignored. Above 40 ^{o}C a truncated mechanism is used that starts from the pR states, since the earlier processes become successively inaccessible at these temperatures. This mechanism includes in addition pB_2 because a second pB phase is identified in the rSVs. 8 observables are faced by 9 free parameters, because this time two scale factors that determine the initial extent of pR_1 and pR_2 have to be included in addition to the 7 rate coefficients. In its full extent, this mechanism is underdetermined and cannot be fit without using additional constraints. Accordingly, we

constrained the initial concentration of pR_1 to 30% of pR_1. In addition we fixed k_6 to 1% of k_4. Both conditions are roughly observed at lower temperatures. With this the fit becomes stable. The temperature dependences of the individual rate coefficients were then fit up to 50 ^{o}C by the transition state equation (TST, Eq. 1, main text) to determine entropy and enthalpy difference to the transition state. Above 50 ^{o}C, PYP starts to deviate from simple thermal activation by occupying more states and rate coefficients extracted at these elevated temperatures were not used for the fit of the TST.

Table S2. Rate coefficients in [1/s] at the temperature range covered (na: not available). T_{jet} is the temperature set by the gas jet controller, and T_{diode} is the temperature measured at the crystal site by the calibrated diode.

T jet	T diode	k_1	k_2	k_3	k_4	k_5	k_6	k_7	k_8	k_9	k_{10}
-40 ^{o}C	235.5 K	2.3×10^{7}	8.3×10^{6}	3.07×10^{6}	63.9	35.9	22.8	3.3	0.05	0	0
-30 ^{o}C	245.1 K	9.9×10^{7}	7.5×10^{7}	8.45×10^{6}	61.0	7.81	25.1	2.7	0.84	0	0
-20 ^{o}C	254.7 K	3.8×10^{8}	1.3×10^{8}	1.29×10^{7}	1.94×10^{2}	7.09	38.2	7.3	0.36	0	0
-15 ^{o}C	259.5 K	4.5×10^{8}	3.3×10^{7}	9.84×10^{6}	2.30×10^{2}	81.4	32.2	26.7	0.77	0	0
-10 ^{o}C	264.3 K	6.0×10^{8}	3.0×10^{8}	1.82×10^{7}	1.68×10^{2}	120	29.9	51.9	2.56	0	0
0 ^{o}C	274.0 K	2.0×10^{9}	6.0×10^{8}	2.67×10^{7}	1.33×10^{3}	122	159	173	8.07	0	0
10 ^{o}C	283.6 K	2.0×10^{9}	9.9×10^{8}	3.18×10^{7}	3.44×10^{3}	802	287	515	14.4	0	0
20 ^{o}C	293.2 K	2.0×10^{9}	1.3×10^{9}	5.34×10^{7}	5.59×10^{3}	2226	198	296	62.9	0	0
25 ^{o}C	293.7 K	3.0×10^{9}	1.8×10^{9}	1.16×10^{8}	4.78×10^{3}	4780	79.0	1100	39.1	0	0
30 ^{o}C	298.5 K	3.0×10^{9}	1.3×10^{9}	7.59×10^{7}	1.16×10^{4}	3440	605	4340	53.3	0	0
40 ^{o}C	308.9 K	3.0×10^{9}	1.5×10^{9}	1.22×10^{8}	1.80×10^{4}	3040	215	3850	190	0	0
[a]50 ^{o}C	318.9 K	- na -	- na -	- na -	6.7×10^{4}	2.8×10^{4}	600	1.8×10^{4}	79.8	250	1.5
[d]60 ^{o}C	328.8 K	- na -	- na -	- na -	6.58×10^{4}	4.28×10^{4}	600	1.0×10^{4}	326	138	24
[d]70 ^{o}C	338.8 K	- na -	- na -	- na -	2.03×10^{5}	1.04×10^{5}	- na -	- na -	97.5	0	0

[a] At 50 ^{o}C and 60 ^{o}C pB_1 decays with k_8+k_9

[d] at T = 70 ^{o}C data quality is poor (see Fig. S5). Therefore, formation and decay of only one pB state is listed.

Microspectrophotometry

Small PYP crystals were crushed between two cover slides which were subsequently sealed with epoxy. The crystalline slurry was probed by a micro-spectrophotometer with a time-resolution of 20 μs. The design of the micro-spectrophotometer will be reported elsewhere (Purwar *et al.*,

2013). Briefly, the light of a 300W Xenon lamp (Ashai spectra) is collected into a tapered fiber. The fiber light is focused to about 200 μm onto the crystalline slurry. The transmitted light is picked up and transported to a Shamrock spectrophotometer equipped with a fast Istar camera (Andor Technology). To avoid bleaching by the intense monitoring Xenon light, a shutter (Thorlabs) restricts exposure to 13 ms. A reaction in the slurry is started by a 449 nm nanosecond laser pulse from the same Opolette II tunable laser which has been used also for the crystallographic experiments. The laser pulse energy density was about 50 $\mu J/mm^2$ at the sample. The absorbance at 449 nm was around one. The laser flashlamp, the Q-switch, the shutters and the fast camera are synchronized by two Stanford Research Systems delay generators. The experiment is controlled by a *MatLab* routine in conjunction with Andor's *Solis* kinetic software. The image intensifier of the Andor detector is time-gated at a time delay Δt after the laser pulse to collect a spectrum. Time-gate pulses are 20 μs for time-delays up to 100 ms and up to 700 μs for time-delays longer than 200 ms. This ensures that at any time delay the signal collected does not suffer from extensive dark noise of the CCD sensor. The photocycle was restarted approximately 25 times to collect an absorption spectrum with a sufficient signal to noise ratio. The temperature was controlled by an Oxford Cryojet HTII (Agilent Technologies) gas stream and determined by a calibrated diode. Difference spectra were generated by subtracting the absorption spectrum collected in the dark from those collected at the time-delays. The time-series of difference spectra (Fig. 6, main text) were analyzed by singular value decomposition in the wavelength range from 410 nm to 530 nm. Absorption values at longer wavelengths only contribute noise and nothing to the kinetics. Although the blue shifted part of the spectra is not included, kinetic phases corresponding to pR and pB can be faithfully distinguished since absorption at each wavelength contains information about the kinetics which is extracted by the

SVD. Relaxation times were determined by fitting exponential functions to the right singular vectors in a similar way as it was done for the time-resolved crystallographic experiments.

Estimation of energy and heat deposition as well as heat diffusion in the PYP crystal due to Laser pulses

The absorption coefficient of crystals is anisotropic (Ng *et al.*, 1995). With unpolarized laser light used in our experiments we can assume that the absorption coefficient of the absorption maximum is equal to that in solution (45,500 cm^2 $mmol^{-1}$), however slightly shifted (3 nm) in the crystal (Fig. S2). The wavelength of the laser used to initiate the reaction is 485 nm. At that wavelength the absorption coefficient is a factor of 10 smaller (see also Fig. S2). The PYP concentration is 96 mmol/L in the crystal. These crystals are exposed to laser light in a geometry shown in Fig. 4A (main text). The penetration depth can be defined as the thickness when 1 a.u. is reached, hence 90% of the photons are absorbed. At 485 nm, 1 a.u. is reached at $d = \frac{1}{\varepsilon \cdot c} = 0.0023 cm$ which is about half of the vertical size of 60 μm of the X-ray beam (Fig. 4B, main text). The laser energy density in the focal spot at the crystal is around 4.0 mJ/mm^2. The focal spot has a diameter of 200 μm. The intersection of the laser beam with the crystal is 200 μm x crystal diameter which is about 120 μm. The fraction of the laser energy that strikes the crystal is therefore 0.2 mm × 0.12 mm × 4.0 mJ/mm^2 = 0.096 mJ. 90 % of this distributes to a volume of 0.2 × 0.12 × 0.023 mm^3 =5.5 × 10^{-10} L, which contains 5.3 × 10^{-8} mmol PYP. With a molecular weight of 14700 g/mol this amount of PYP has a mass of 8 x 10^{-10} kg. If we assume that half of the laser light is dissipated as heat into the vibrational modes of motion and half of it is stored in an energy rich chromophore configuration (Martin *et al.*, 1983, van Brederode *et al.*, 1995), we can estimate the adiabatic temperature rise from the fraction of the heat dissipated into

the vibrational modes. With a heat capacitance of a typical protein of 5 kJ kg^{-1} K^{-1} (Miyazaki *et al.*, 2000), we would expect an increase of $0.5\times 0.9\times \frac{0.096\times 10^{-6}\,kJ}{8\times 10^{-10}\,kg\times 5kJ\,kg^{-1}K^{-1}}=11K$, which is about the typical temperature step of ~10 K we used. The energy stored in the chromophore configuration is gradually released. The total heat is diffusing out of the illuminated and excited volume. Since the penetration depth of the laser is much smaller than the crystal diameter, and the face of the crystal is in contact with the temperature controlled capillary surface we have a two dimensional heat diffusion problem with the heat escaping to the left and right into the crystal volume and into the capillary wall and into the bulk of the crystal volume below the illuminated volume. The characteristic time for the heat to diffuse out of that volume is (Carslaw & Jaeger, 1959)

$$\tau=\frac{1}{\kappa\pi^2\left(\frac{1}{a^2}+\frac{1}{b^2}\right)},$$

with κ the thermal diffusivity and a and b the size of the two-dimensional box shown in Fig. 2 (main text). With a=0.2 mm, b=0.023 mm, and assuming that κ of the protein crystal equals to that of water being 0.143 mm^2/s, we estimate a characteristic time of roughly 0.5 ms for the heat to diffuse out of the laser illuminated volume. Moffat et al. (Moffat *et al.*, 1992) estimate heat diffusion times that are much longer based on calculations with larger illuminated volumes.

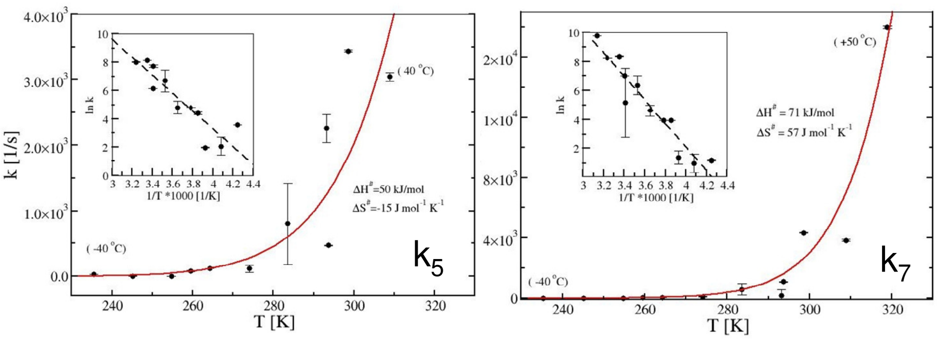

Figure S1. Temperature dependence of rate coefficients k_5 and k_7. Decay of pR_1 to pB_1 (k_5) and pG (k_7). Black spheres: rate coefficients, red line: fit by the transition state equation. Entropy and enthalpy of the barriers are listed. Inserts: corresponding Arrhenius plots.

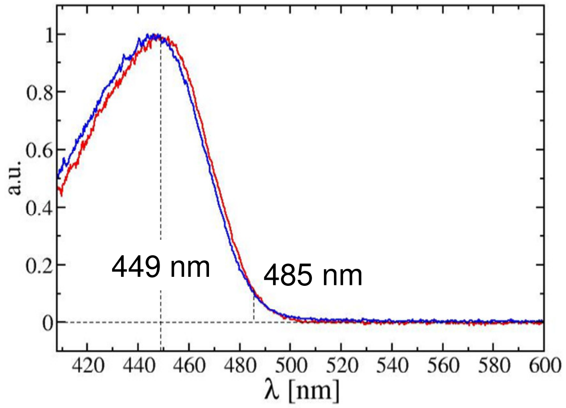

Figure S2. Typical PYP absorption spectrum as measured by the micro-spectrophotometer without laser illumination. Blue: solution, Red: crystalline slurry. Spectra normalized. Absorption maximum of crystal is shifted by ~ 3 nm as compared to solution. The absorption at 485 nm is a factor of ~10 smaller than at 449 nm.

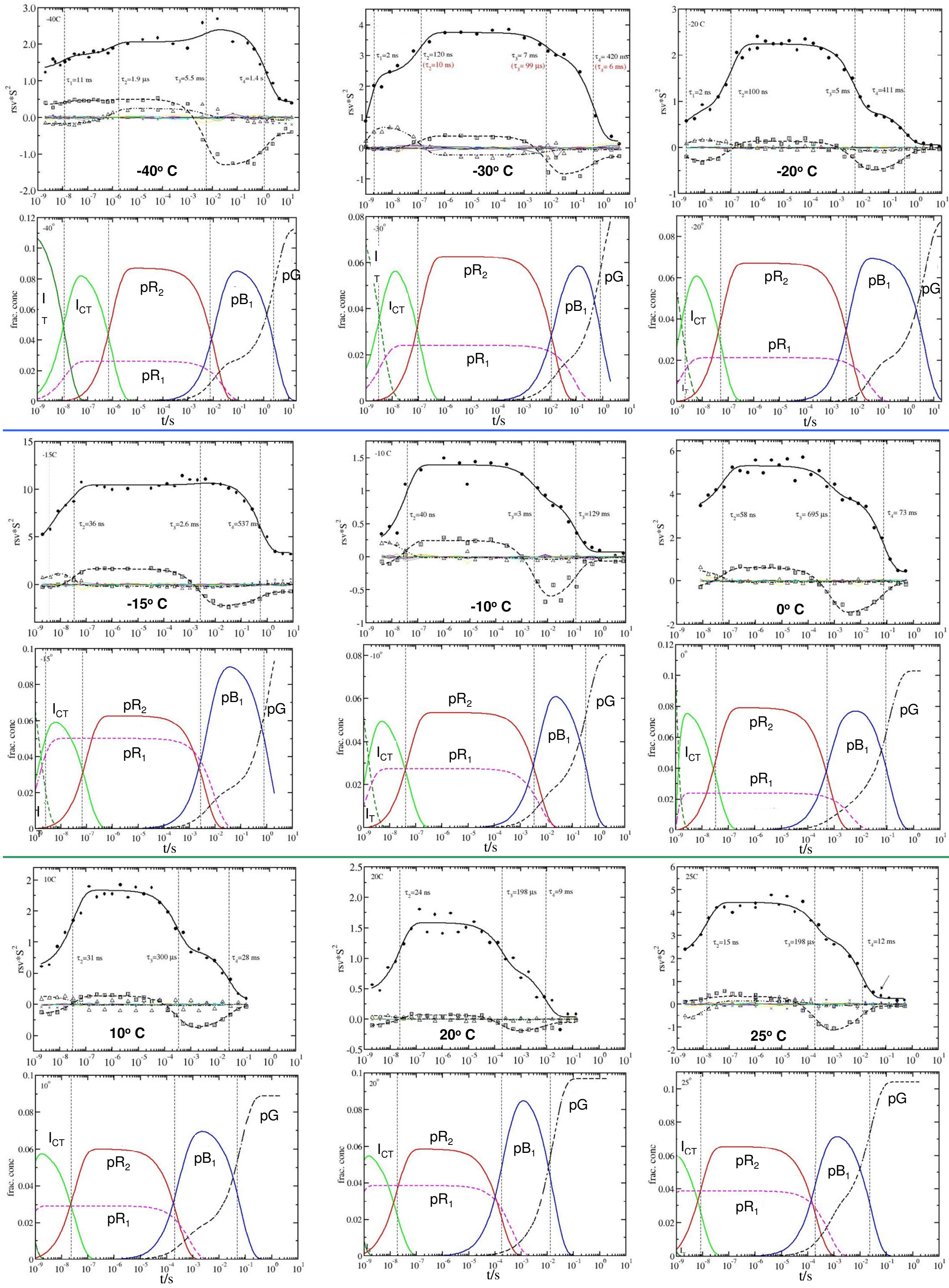

-40° C
τ1=11 ns
τ2=1.9 µs
τ3=5.5 ms
τ4=1.4 s
-30° C
τ1=2 ns
τ2=120 ns
(τ2=10 ns)
τ3= 7 ms
(τ3= 99 µs)
τ4= 420 ms
(τ4= 6 ms)
-20° C
τ1=2 ns
τ2=100 ns
τ3=5 ms
τ4=411 ms
-15° C
τ2=36 ns
τ3=2.6 ms
τ4=537 ms
-10° C
τ2=40 ns
τ3=3 ms
τ3=129 ms
0° C
τ2=58 ns
τ3=695 µs
τ4= 73 ms
10° C
τ2=31 ns
τ3=300 µs
τ4=28 ms
20° C
τ2=24 ns
τ3=198 µs
τ4=9 ms
25° C
τ2=15 ns
τ3=198 µs
τ4=12 ms
rsv*S²
frac. conc
t/s
I
T
ICT
pR2
pR1
pB1
pG

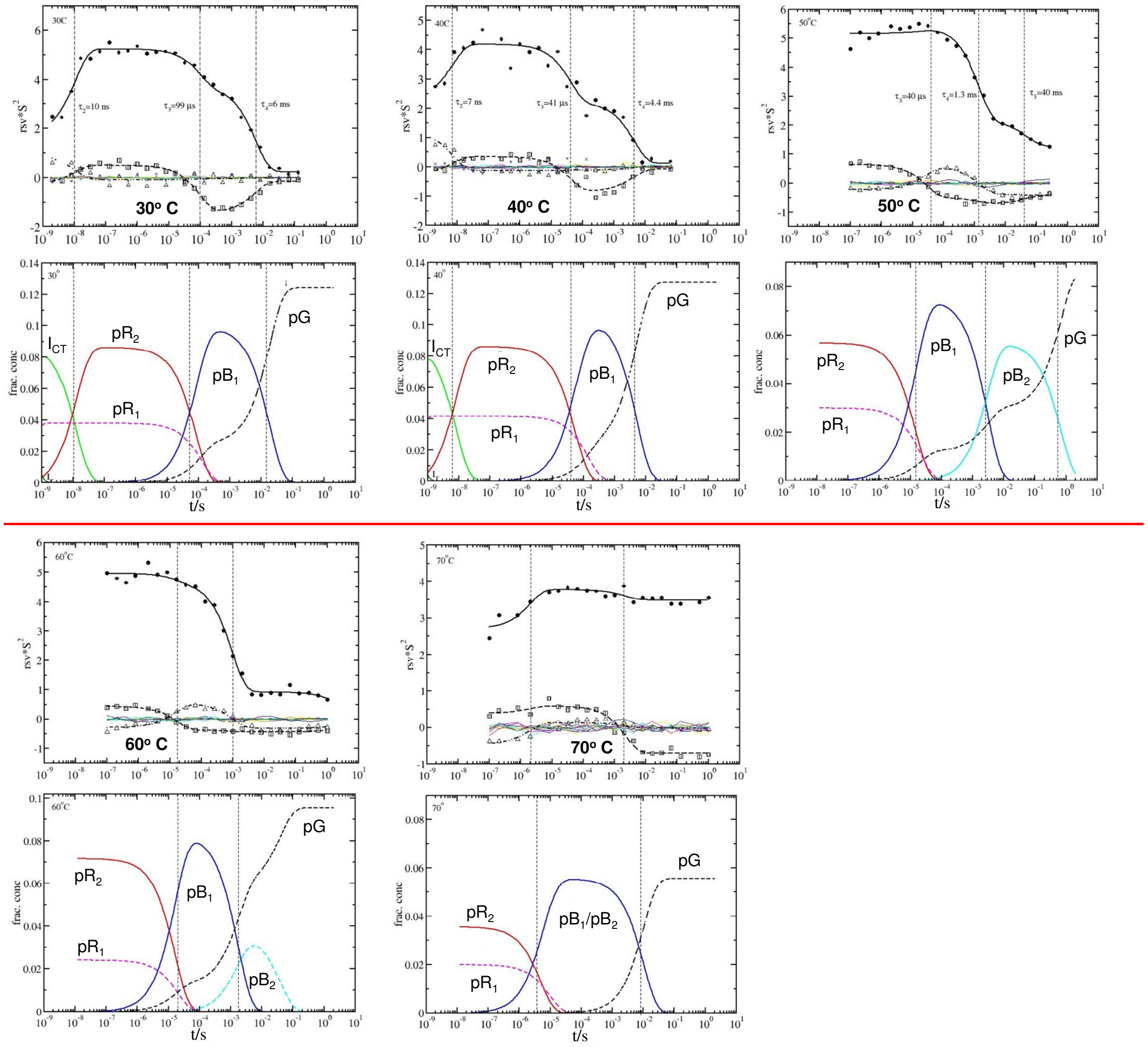

Figure S3. Right singular vectors (upper panels) as extracted at the 14 temperatures. Symbols denote the components of the right singular vectors multiplied with the square of the corresponding singular value. Black lines are from the global fit of a sum of exponentials. Solid spheres and black solid lines: first right singular vectors, open squares and black dashed lines: second right singular vectors, open triangles and black dashed dotted lines: third right singular vectors. Colored lines: less significant right singular vectors 4-15. Corresponding time-dependent fractional concentrations are shown in the panels below. Dark green: concentrations of I_T. The dashed dark-green color is used when I_T decays within the pulse duration of the laser. Green: concentrations of I_{TC}. Red: concentrations of pR_2. Dashed magenta: concentrations of pR_1, this pathway is a minor pathway. Blue: concentrations of pB. Light blue: concentrations of pB_2. Dashed black: recovery of pG. Black vertical lines in upper and lower panels: comparison of relaxation times from the singular value decomposition and from posterior analysis. At T$\geq$ 60 ^{o}C (below the thick red line), the offset in the rSV becomes excessively large, which indicates poor and noisy data. A kinetic analysis becomes challenging.

Literature

Borgstahl, G. E., Williams, D. R. & Getzoff, E. D. (1995). *Biochemistry* **34**, 6278-6287.

Carslaw, H. S. & Jaeger, J. C. (1959). *Conduction Heat in Solids*, 2nd edition ed. Oxford: Clarendon Press.

Cornish-Bowden, A. (2012). *Fundamentals of Enzyme Kinetics*, 4 edition ed. Wiley-VCH.

Graber, T., Anderson, S., Brewer, H., Chen, Y. S., Cho, H. S., Dashdorj, N., Henning, R. W., Kosheleva, I., Macha, G., Meron, M., Pahl, R., Ren, Z., Ruan, S., Schotte, F., Srajer, V., Viccaro, P. J., Westferro, F., Anfinrud, P. & Moffat, K. (2011). *J Synchrotron Radiat* **18**, 658-670.

Ihee, H., Rajagopal, S., Srajer, V., Pahl, R., Anderson, S., Schmidt, M., Schotte, F., Anfinrud, P. A., Wulff, M. & Moffat, K. (2005). *Proceedings of the National Academy of Sciences of the United States of America* **102**, 7145-7150.

Jung, Y. O., Lee, J. H., Kim, J., Schmidt, M., Moffat, K., Srajer, V. & Ihee, H. (2013). *Nature chemistry* **5**, 212-220.

Khoroshyy, P., Der, A. & Zimanyi, L. (2013). *Journal of photochemistry and photobiology. B, Biology*.

Martin, J. L., Migus, A., Poyart, C., Lecarpentier, Y., Astier, R. & Antonetti, A. (1983). *Proceedings of the National Academy of Sciences of the United States of America* **80**, 173-177.

Miyazaki, Y., Matsuo, T. & Suga, H. (2000). *Journal of Physical Chemistry B* **104**, 8044-8052.

Moffat, K., Chen, Y., Ng, K. M., Mcree, D. & Getzoff, E. D. (1992). *Philos T R Soc A* **340**, 175-189.

Ng, K., Getzoff, E. D. & Moffat, K. (1995). *Biochemistry* **34**, 879-890.

Purwar, N., Tenboer, J., Tripathi, S. & Schmidt, M. (2013). *International Journal of Molecular Sciences* **14**, 17.

Rajagopal, S., Anderson, S., Srajer, V., Schmidt, M., Pahl, R. & Moffat, K. (2005). *Structure* **13**, 55-63.

Schmidt, M. (2008). *Structure Based Enzyme Kinetics by Time-Resolved X-ray crystallography, in: Ultrashort Laser Pulses in Medicine and Biology*. Germany: Berlin ; New York : Springer, c2008.

Schmidt, M., Graber, T., Henning, R. & Srajer, V. (2010). *Acta crystallographica. Section A, Foundations of crystallography* **66**, 198-206.

Schmidt, M., Ihee, H., Pahl, R. & Srajer, V. (2005). *Methods Mol Biol* **305**, 115-154.

Schmidt, M., Nienhaus, K., Pahl, R., Krasselt, A., Anderson, S., Parak, F., Nienhaus, G. U. & Srajer, V. (2005). *Proceedings of the National Academy of Sciences of the United States of America* **102**, 11704-11709.

Schmidt, M., Pahl, R., Srajer, V., Anderson, S., Ren, Z., Ihee, H., Rajagopal, S. & Moffat, K. (2004). *Proceedings of the National Academy of Sciences of the United States of America* **101**, 4799-4804.

Schmidt, M., Rajagopal, S., Ren, Z. & Moffat, K. (2003). *Biophysical journal* **84**, 2112-2129.

Steinfeld, J. I., Francisco, J. S. & Hase, W. L. (1985). *Chemical Kinetics and Dynamics*, 2 edition ed. Prentience Hall.

Tripathi, S., Srajer, V., Purwar, N., Henning, R. & Schmidt, M. (2012). *Biophysical journal* **102**, 325-332.

van Brederode, M. E., Gensch, T., Hoff, W. D., Hellingwerf, K. J. & Braslavsky, S. E. (1995). *Biophysical journal* **68**, 1101-1109.

van Stokkum, I. H., Larsen, D. S. & van Grondelle, R. (2004). *Biochimica et biophysica acta* **1657**, 82-104.

Yeremenko, S., van Stokkum, I. H., Moffat, K. & Hellingwerf, K. J. (2006). *Biophysical journal* **90**, 4224-4235.
